# A rare association of pheochromocytoma, paraganglioma, and pituitary adenoma (3PA): A case report and literature review

**DOI:** 10.1097/MD.0000000000038928

**Published:** 2024-08-09

**Authors:** Rahem Rahmati, Elahe Meftah, Hamed Hamidi, Fatemeh Esfahanian

**Affiliations:** a Students Research Committee, Shahrekord University of Medical Sciences, Shahrekord, Iran; b Students’ Scientific Research Center, Tehran University of Medical Sciences, Tehran, Iran; c Department of Endocrinology, Vali-Asr Hospital, Imam Khomeini Hospital Complex, Tehran University of Medical Sciences, Tehran, Iran.

**Keywords:** 3P association, case report, MYC-associated factor X, paraganglioma, pheochromocytoma, pituitary adenoma, succinate-dehydrogenase

## Abstract

**Rationale:**

3P association (3PA) is a rare condition with co-occurrence of pituitary adenoma and pheochromocytoma/paraganglioma. There have been less than a hundred documented cases of 3PA, which can be sporadic or related to genetic mutations. The present case report describes the first Iranian patient with 3PA and a 90th case of 3PA in the available literature.

**Patient concerns and interventions:**

A 36-year-old Caucasian male was admitted with headache and sudden increase in blood pressure. An abdominal CT scan revealed a retroperitoneal mass posterior to the inferior vena cava, later removed and diagnosed as a pheochromocytoma. Four years later, he noticed occasional mild headaches and a painless mass on the right side of his neck. The ultrasonography evaluations suggested a carotid body tumor, which was surgically removed. About a month after his second surgery, the severity of the patient’s headaches worsened, and he developed right homonymous hemianopia. A brain MRI showed a mass in favor of macroadenoma, craniopharyngioma, or meningioma, and elevated prolactin level led to the diagnosis of macroprolactinoma.

**Diagnoses:**

Based on the provided history, this patient was diagnosed with 3PA, and a genetic study identified a positive succinate-dehydrogenase-complex subunit b mutation, possibly linked to his family history of carotid body tumor.

**Outcomes:**

He has remained symptom-free during his visits every 3 months.

**Lessons:**

The number of cases diagnosed with 3PA worldwide is increasing. Using clinical and genetic assessments, we can timely diagnose and adequately monitor individuals with or at risk of 3PA.

## 1. Introduction

3P association (3PA) is a rare condition defined as the co-occurrence of endocrine neoplasms, including pituitary adenoma (PA) and chromaffin cell neoplasia in forms of pheochromocytoma/paraganglioma (Pheo/PGL).^[1]^ To date, only 89 patients with 3PA have been documented.^[2–4]^ 3PA may be sporadic or have underlying genetic mutations, including mutations in succinate dehydrogenase genes. In light of earlier suggestions, the genetic evaluation can strengthen the previously discovered correlations, uncover new information and possible novel genetic mutations, and further clarify the differences between 3PA and other endocrine neoplasia syndromes.^[5]^ To the best of our knowledge, the present case report describes the first Iranian patient with 3PA and a 90th case of 3PA in the available literature, including clinical and genetic characteristics.

## 2. Case presentation

In April 2016, a 36-year-old Caucasian male presented at the emergency department with headache and a new sudden spike in blood pressure following an anger episode. He had a history of dyspepsia for which he took omeprazole 20 mg daily and was otherwise healthy without any positive habitual or past surgical history. His family history was positive for primary hypertension in his mother and his parents had consanguineous marriage. In the physical examination, he had tachycardia, flushing, and a systolic blood pressure equal to 250 mm Hg. No other positive finding was noted in his examination. Once his blood pressure was stabilized, he was advised to an outpatient visit with a nephrologist for further evaluation.

During the evaluations for secondary hypertension, an abdominal CT scan demonstrated a 50 * 40 mm retroperitoneal mass posterior to the inferior vena cava (IVC), with adhesions to the right renal artery, renal vein, and adrenal gland. Serum urea, creatinine, and urinary metanephrine levels were within normal values. Nevertheless, elevated levels of urinary norepinephrine (361 mcg/24 hours, reference range: 15–80 mcg/24 hours) and normetanephrine (850 mcg/24 hours, reference range: 0–600 mcg/24 hours) were found in the 24-hour urine. With the diagnosis of pheochromocytoma, the patient’s blood pressure was controlled by phenoxybenzamine 10 mg daily for 18 months, until the patient consented to the surgery. Later, in 2018, he underwent open right total adrenalectomy with the release of IVC and right renal artery and vein. He was recovered well. The histopathology findings proved the diagnosis of pheochromocytoma.

The patient remained symptom-free until 2020, when he began experiencing occasional mild headaches without any accompanying symptoms, which he initially ignored. Later the same year, he accidentally detected a painless mass on the right side of his neck. The ultrasonography evaluations revealed a noncalcified hypervascular hypoechoic solid mass measuring 36 * 30 * 22 mm with smooth margins in the bifurcation of the right common carotid artery, suggesting a carotid body tumor. Later the same month, he underwent resection of the carotid body tumor and cervical lymph nodes, and the pathologic evaluation proved the suggested diagnosis. He recovered without notable problem. Interestingly, his sister had also undergone surgery to remove a carotid body tumor just 2 months before his procedure.

About a month after his second surgery, the severity of the patient’s headaches worsened, which led to a referral to a neurosurgeon for further evaluation. He had also experienced blurred vision in his right eye since the onset of his headaches in 2020, which later perimetry revealed right homonymous hemianopia. A brain MRI revealed a 35 * 32 * 27 mm mass arising from the Sella turcica with extra-axial origin and attachment to sphenoid bone, with severe and homogenous enhancement after gadolinium injection, suggesting macroadenoma, craniopharyngioma, or meningioma. Subsequent laboratory tests demonstrated prolactin level > 200 ng/mL (reference range: 1.9–18.3 ng/mL), leading to a diagnosis of macroprolactinoma. Following the diagnosis of macroprolactinoma, the patient was prescribed Cabergoline, with an initial dose of 0.5 mg that gradually escalated to a total weekly dose of 3 mg. This treatment significantly improved his symptoms, including his headaches and blurred vision, and his prolactin level decreased significantly (150 microIU/L (normal: <425 mIU/L) in the latest evaluation 30 months after the initiation of Cabergoline. Additionally, the adenoma measured 21 * 25 * 22 mm in the last follow-up brain MRI.

Based on the provided presentations, this patient was diagnosed with 3PA, which includes pheochromocytoma, paraganglioma, and prolactinoma (PA). Given the patient’s positive family history and the potential involvement of underlying genetic factors in this association, a genetic study was conducted in December 2021, indicating a positive succinate-dehydrogenase-complex subunit b (SDHB) mutation (c.688 > T p. Arg230Cys variant). No mutations were detected in the other 48 assessed genes, including multiple endocrine neoplasia 1 (MEN1), RET, SDHA, SDHC, SDHD, and Von Hippel-Lindau (VHL).

The patient currently manages his dyspepsia with pantoprazole and takes propranolol for palpitations, Migraphar^®^ for headaches, sodium valproate for preventing seizures, vitamin D, and calcium. He has remained symptom-free during his routine appointments, which take place every 3 months.

## 3. Discussion

The prevalence of symptomatic PA is approximately one in every 1000 to 1500 people. Only 5% of PAs occur within families, with mutations commonly observed in MEN1 and aryl hydrocarbon receptor interacting protein (AIP) genes.^[2]^ Pheo/PGL tumors are even less common, with an estimated prevalence ranging from 1 in 2500 to 1 in 6667. It is believed that around 30% to 40% of cases with Pheo/PGL are associated with hereditary conditions, mostly germline mutations in SDH genes, while somatic mutations are identifiable in 25% to 30% of nontumors.^[2]^ According to the prevalence data, the coincidental chance of an individual having both PA and Pheo/PGL ranges from 1 in 2.5 to 8.5 million in the general population.^[5]^

Tumor DNA analysis has led to the discovery of numerous predisposing genes of Pheo/PGL and PA, initiating the transition from association to causality over the past decade.^[6]^ In this regard, Denes et al^[5]^ proposed several potential explanations for the simultaneous occurrence of these tumors: a mutation in a gene associated with Pheo/PGL also leads to the development of PA, as seen in cases with SDHx mutation; a mutation in a familial PA gene also results in the formation of Pheo/PGL; digenic disease; where 2 genetic abnormalities coexist; the presence of a single, possibly novel gene that is responsible for the development of Pheo/PGL and PA; ectopic hypothalamic hormone-secreting adrenal tumors that cause extension of the pituitary gland, mimicking the symptoms of PA; pure coincidence occurring by chance.

Currently, there is widespread acceptance that 3PA represents a newly inherited predisposition to multiple endocrine tumors resulting from SDHx defects.^[3]^ Mutations in SDH genes suppress prolyl hydroxylases in the cytosol, stabilizing and activating hypoxia-inducible factor-alpha. This process promotes tumor development by activating angiogenesis, glucose metabolism, and cell survival.^[2]^ SDH mutations result in different behaviors of the neoplasms in the 3PA compared to the sporadic cases. Individuals with genetic mutations in SDH, particularly the beta subtype, tend to exhibit multiple and more invasive tumors and an earlier onset of Pheo/PGL.^[1]^ Additionally, SDH-related pituitary tumors exhibit a distinctive trait marked by intracytoplasmic vacuoles. While over half of the cases are prolactinomas, other types of pituitary tumors are also observed.^[7–9]^

Xekouki et al^[3]^ found that in familial cases of PGLs, SDHx mutations were present in 62.5% to 75% of the cases documented, while in sporadic cases of 3PAs, no SDHx defects were identified. Considering the existing literature, many cases with 3PA do not have an uncovered germline mutation, indicating that additional genes related to 3PAs are yet to be discovered. 3PA negative for SDHx germline mutations may be attributed to other genes like MEN1, RET, VHL, or suppressor MYC-associated factor X (MAX). Epigenetic alterations, mutations in modifier genes, mosaicism, or somatic mutations may also be contributive.^[2,3]^ Nevertheless, some studies consider 3PA negative for all SDH subunits as a new variation of MEN syndrome with an unknown genetic mutation.^[2]^

To the best of our knowledge and considering our case, 90 cases with 3PA have been reported so far. Among them, 35 (38.88%) individuals had a genetic mutation associated with a predisposition to Pheo/PGLs or PAs. Twenty-seven patients (30%) had either personal or family histories indicative of a hereditary endocrine syndrome, while 40 (44.44%) cases were isolated. Family history information was unknown for 23 (25.55%) of the patients with 3PA. Seventeen patients (18.88%) had both a confirmed genetic mutation and a family history. Most cases exhibited SDHx defects (22/61 cases identified, 36.06%), followed by MEN1 and MAX as the most common associated genes.

Following diagnosis, Xekouki et al^[3]^ recommend further evaluation and ongoing monitoring in cases with 3PA. They suggest genetic screening for SDHx mutations in patients with a positive family history of Pheo/PGL. If the screening results are positive, it is advisable to offer the screening to other family members as well. Annual follow-up and clinical evaluation are recommended in the case of a positive screening finding. Conversely, if the patient does not have a positive family history of Pheo/PGL, the assessment should focus on MEN1/MEN2, menin/RET, and then SDHx, MAX, or other genes. Specifically, when a MAX mutation is present, comprehensive medical history and physical examination are crucial for all carriers of mutations to screen for additional tumors linked to MAX mutations, including PA and renal cancers.^[3]^

## 4. Conclusion

The global incidence of diagnosed 3PA cases is on the rise. While research on patients with 3PA is limited, improved monitoring and diagnosis through genetic assessment and analysis of clinical characteristics can provide valuable insights for early identification of at-risk individuals and implementing screening and possible gene-therapy preventive strategies. Where genetic testing is not available, additional attention to the clinical presentation and considering the possibility of an association is pivotal for patient management.

## Author contributions

**Visualization:** Rahem Rahmati.

**Writing – original draft:** Rahem Rahmati, Hamed Hamidi.

**Writing – review & editing:** Rahem Rahmati, Elahe Meftah, Fatemeh Esfahanian.

**Data curation:** Elahe Meftah.

**Investigation:** Elahe Meftah, Hamed Hamidi.

**Conceptualization:** Fatemeh Esfahanian.

**Resources:** Fatemeh Esfahanian.
